# Potential Effects of Soy Isoflavones on the Prevention of Metabolic Syndrome

**DOI:** 10.3390/molecules26195863

**Published:** 2021-09-27

**Authors:** Kazuo Yamagata, Yukio Yamori

**Affiliations:** 1 Department of Food Bioscience & Biotechnology, College of Bioresource Science, Nihon University (UNBS), Fujisawa 282-8510, Japan; 2 Institute for World Health Development, Mukogawa Women’s University, Nishinomiya 663-8143, Japan; yamori@cardiacstudy.com

**Keywords:** soy, isoflavones, genistein, daidzein, metabolic syndrome

## Abstract

Isoflavones are polyphenols primarily contained in soybean. As phytoestrogens, isoflavones exert beneficial effects on various chronic diseases. Metabolic syndrome increases the risk of death due to arteriosclerosis in individuals with various pathological conditions, including obesity, hypertension, hyperglycemia, and dyslipidemia. Although the health benefits of soybean-derived isoflavones are widely known, their beneficial effects on the pathogenesis of metabolic syndrome are incompletely understood. This review aims to describe the association between soybean-derived isoflavone intake and the risk of metabolic syndrome development. We reviewed studies on soy isoflavones, particularly daidzein and genistein, and metabolic syndrome, using PubMed, ScienceDirect, and Web of Science. We describe the pathological characteristics of metabolic syndrome, including those contributing to multiple pathological conditions. Furthermore, we summarize the effects of soybean-derived daidzein and genistein on metabolic syndrome reported in human epidemiological studies and experiments using in vitro and in vivo models. In particular, we emphasize the role of soy isoflavones in metabolic syndrome-induced cardiovascular diseases. In conclusion, this review focuses on the potential of soy isoflavones to prevent metabolic syndrome by influencing the onset of hypertension, hyperglycemia, dyslipidemia, and arteriosclerosis and discusses the anti-inflammatory effects of isoflavones.

## 1. Introduction

Several studies have shown that soy and isoflavone intake is inversely associated with the incidence of various chronic diseases [1,2]. The potential of soy in the prevention of obesity [3], dyslipidemia [3], hyperglycemia [4], and hypertension [5] has been reported. Recently published systematic reviews have shown that the intake of soy products effectively improves the lipid profiles and glycemic parameters in patients with metabolic syndrome [6]. Soybean is rich in proteins and has a well-balanced amino acid profile. Furthermore, it contains various other nutritive components, such as dietary fiber and isoflavonoids, with beneficial physiological properties [7]. Multiple isoflavones, such as daidzein and genistein, are present in soybean. Since these isoflavones have a structure similar to that of estrogen, they are often referred to as phytoestrogens and have been shown to be involved in multiple estrogen-related bioactivities [8].

Isoflavones are classified into one of the polyphenol subgroups, and have multiple components. The known isoflavones genistein, glycitein, daidzein, formononetin, and biochanin A are present in both aglycone and glycoside forms in plants, such as soybean [8]. Equol, a daidzein metabolite formed by the gut microbiota, is often classified as an isoflavone; however, since equol is not a natural plant compound, it is not strictly an isoflavone [9]. Soybean and processed soybean foods are the primary dietary sources of isoflavones, such as daidzein, genistein, and glycitein, for humans. Besides soybean, chickpeas, fruits, vegetables, and nuts are also dietary sources of isoflavones [10]. Red clover, a type of grass that contains the isoflavones formononetin and biochanin A, is a source of isoflavones for animals, including livestock [11].

Metabolic syndrome, a condition characterized by hypertension, hyperglycemia, and dyslipidemia along with visceral obesity, promotes arteriosclerosis and increases the risk of myocardial infarction and stroke, which are associated with a high mortality rate [12,13]. The incidence of arteriosclerosis is significantly high in patients with metabolic syndrome [14], and the incidences of high-mortality heart disease and stroke are greater in individuals with metabolic syndrome than in those with the individual contributing conditions [15]. The number of metabolic syndrome-related deaths has increased in Europe [16], rendering metabolic syndrome a major global healthcare concern [17]. The relationship between metabolic syndrome and obesity is particularly important, and the production of adipocytokines, which contribute to disease development, has been shown to increase in the significantly enlarged adipocytes of individuals with obesity [18]. In addition, obesity induces the production of reactive oxygen species (ROS) and strongly promotes the development of metabolic syndrome [19,20]. In particular, increased oxidative stress and inflammation are associated with obesity and play an important role in the development of metabolic syndrome-induced heart disease and stroke [21]. Moreover, persistent mild chronic inflammation and immune system activation are closely associated with obesity-induced insulin resistance and the etiology of type 2 diabetes [22]. The regulation of metabolic syndrome-associated chronic inflammation and the prevention of each condition that contributes to metabolic syndrome may help manage these diseases with high mortality rates.

Isoflavone intake can reverse metabolic disorders, such as hyperlipidemia, hypertension, and non-alcoholic fatty liver disease (NAFLD) [23]. In addition, phytoestrogens have been reported to help prevent metabolic syndrome [24]. This review summarizes the results of a wide range of basic and clinical studies and assesses the characteristics of multiple metabolic syndrome-inducing diseases and the relationship between metabolic syndrome and soy isoflavone intake. Furthermore, the effects of soy isoflavones on metabolic syndrome and the underlying mechanisms of action are discussed. To this end, a literature research was conducted using PubMed, ScienceDirect, and Web of Science to identify studies that reported the effects on daidzein and genistein on obesity, dyslipidemia, hyperglycemia, and hypertension, which are conditions that lead to metabolic syndrome. In particular, the literature was selected by focusing on the pathological characteristics of metabolic syndrome and the importance of the potential action and role of isoflavones on metabolic syndrome. The effects of isoflavones on metabolic syndrome and the potential of isoflavones for preventing metabolic syndrome are also discussed.

## 2. Soybean Components and Isoflavone Characteristics

### 2.1. Soybean Components

Soybean is rich in proteins, carbohydrates, and fats, with approximately 36% protein content [25], which is considerably greater than the average protein content of other legumes (20–25%) (Table 1). At approximately 30 g carbohydrates per 100 g, the carbohydrate content in soybean is highest after its protein content. However, the carbohydrate content of soybean is significantly lower than that of other legumes. The low carbohydrate content and high protein content is a unique characteristic of soybean, differentiating it from other legumes. In addition, soybean has been found to be a source of good quality protein, with amino acid scores similar to those of foods derived from animal sources, such as eggs and milk [26]. Furthermore, the lipid content in soybean (20%) is considerably higher than that in other legumes. The lipids present in soybeans are composed of approximately 2.7% saturated fatty acids, 4.4% monounsaturated fatty acids, and 11.2% polyunsaturated fatty acids [27]. Whereas the linoleic acid content is the highest, other fatty acids including oleic acid, palmitic acid, and stearic acid, are also present. In soybean, most fatty acids are present as triacylglycerols [28]. Legumes are also an excellent source of dietary fiber. The dietary fiber content of soybean is approximately 9.9%, which is approximately the same as that of other legumes. Previous studies have shown that one legume meal can provide approximately 2–4 g of soluble and insoluble fiber [29]. In addition, the levels of micronutrients, such as iron and calcium, are high in legumes, which makes them an excellent source of these micronutrients. In particular, soybean has a higher iron and calcium content compared to that of other legumes (Table 1) [25]. Furthermore, the bioavailability of calcium from soybean and soy-based foods was also shown to be as high as that from milk [30].

### 2.2. Dietary Sources of Isoflavones

Although soybean is the main dietary source of isoflavones for humans, isoflavones are also found in legumes, such as soybean, common beans, navy beans, and red clover [25]. Genistein and daidzein are the primary isoflavones present in soybean, while glycitein is present at low levels [28]. Soybeans and processed soybean foods have an adequate isoflavone content (Table 2) [25]. The actual isoflavone content in roasted soybean flour is 198.95 mg/100 g (edible portion) of the total isoflavone content. Genistein, daidzein, and glycitein are present at 99.27 mg/100 g (edible portion), 98.75 mg/100 g (edible portion) and 16.40 mg/100 g (edible portion), respectively. Hence, the glycitein content of roasted soybean flour was found to be substantially lower than the genistein and daidzein contents [31].

### 2.3. Isoflavone Types and Structures

**Figure 1:**
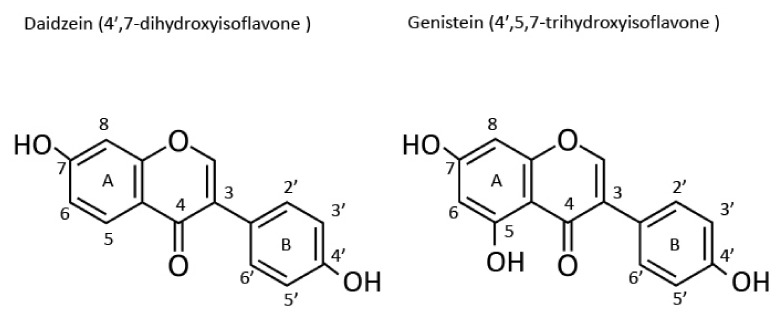
Structures of daidzein and genistein.

Isoflavones form a subgroup of flavonoids, which are a type of polyphenols. All polyphenols classified as flavonoids have flavonoid-like structures [32]. Isoflavones have two benzene rings (A and B) linked by a heterocyclic pyran residue. The benzene ring B is attached to the carbon at position 3 of the heterocyclic pyran residue (Figure 1) [28]. In contrast, in all flavonoids except isoflavones, the benzene ring B is attached to the carbon at position 2 of the heterocyclic pyran. This structural difference clearly distinguishes isoflavones from flavonoids [28]. Furthermore, the structural difference between daidzein and genistein depends on the presence of a hydroxyl group at position 5 of the A ring in genistein, part of an internal hydrogen bond, which is absent in daidzein. Genistein is 4′,5,7-trihydroxyisoflavone, daidzein is 4′,7-dihydroxyisoflavone, and glycitein is 7,4′-dihydroxy-6-methoxyisoflavone. Glycosides with a β-glycosidic bond between the sugar and the carbon at position 7 of the A ring of genistein and daidzein are referred to as genistin and daidzin, respectively [28]. The initial popularity of isoflavones was based on their beneficial effects, which were mediated via their estrogen-like activity and attributed to their structure. However, previous in vitro and in vivo studies have shown that isoflavones exert weak effects and exhibit activities that are 1 × 10^−4^ to 1 × 10^−3^ times those of 17β-estradiol [28,33]. The effects of isoflavones subtly differ based on the differences in their structures.

### 2.4. Absorption of Isoflavones in the Human Body

Several studies have been conducted on the bioavailability of daidzein in humans and rats. When healthy volunteers ingested soymilk, the total serum isoflavone concentration gradually increased to 24.7 ± 10 mg/L at 6 h after ingestion [34]. In another study in humans, the blood daidzein concentration increased 1 h after the ingestion of daidzein at 1 mg/kg body weight, became steady at 4.5 h, and then gradually decreased. The daidzein concentration in the blood and urine significantly increased (by approximately three- to six-fold) after daidzein ingestion compared to that before daidzein ingestion, suggesting that ingested isoflavones are absorbed by the body [35].

## 3. Metabolic Syndrome: Definition and Pathological Characteristics

### 3.1. Definition of Metabolic Syndrome

The definition of metabolic syndrome was first provided by the World Health Organization (WHO) in 1998 [36]. According to the WHO, several factors, in addition to diabetes, impaired fasting glucose levels, or insulin resistance, are to be considered while defining metabolic syndrome. The European Group for the Study of Insulin Resistance indicated that more an appropriate term than “insulin resistance” should be used in the definition of metabolic syndrome [37]. This group suggested adding “increase in waist circumference” as a term to represent the element of abdominal obesity. The International Diabetes Federation defines metabolic syndrome based on the presence of central obesity along with two or more cardiovascular risk factors [38]. The National Cholesterol Education Program Adult Treatment Panel III 6 simplified the criteria for defining metabolic syndrome, making it widely accessible to practitioners [39]. It has been well-established that metabolic syndrome is a pathological condition characterized by chronic, systemic, and mild inflammatory and oxidative conditions, as well as clusters of three or more independent risk factors for heart disease [40]. In general, metabolic syndrome involves abdominal obesity, hyperglycemia/insulin resistance, hypertriglyceridemia, high levels of low-density lipoprotein (LDL), low levels of high-density lipoprotein (HDL) cholesterol, and high blood pressure. Each contributing disease is treated as a separate pathological condition intricately related to the onset and progression of the syndrome [41].

### 3.2. Pathological Characteristics of Metabolic Syndrome

**Figure 2:**
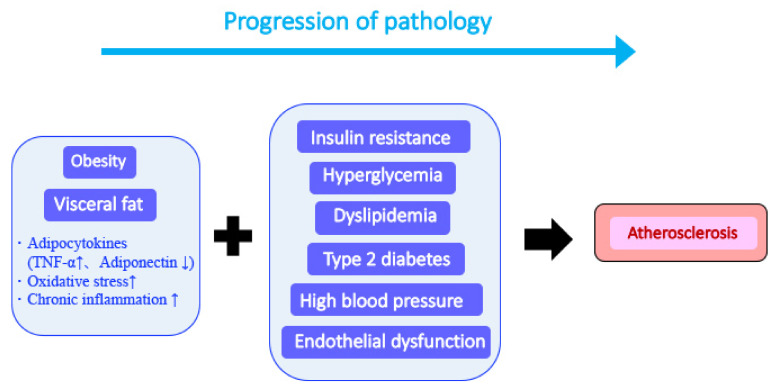
Progression from metabolic syndrome to arteriosclerosis. Obesity causes the excessive accumulation of visceral fat. Increased secretion of inflammatory cytokines such as tumor necrosis factor alpha from visceral fat and the strong induction of various diseases collectively cause arteriosclerosis.

An interplay between visceral steatosis, insulin resistance, dyslipidemia, and hypertension leads to metabolic syndrome progression [42]. Visceral fat accumulation induces changes in the production of various adipocytokines (Figure 2). For example, an increase in tumor necrosis factor alpha (TNF-α) levels and a decrease in adiponectin levels are early signs of significantly increased inflammatory stimulation. These phenomena induce chronic inflammation, exacerbate various conditions, and accelerate arteriosclerosis development. A metabolic syndrome hallmark is the presence of multiple complex conditions resulting from metabolic disorders and the association with hyperglycemia, dyslipidemia, hypertension, and visceral obesity [43]. Another characteristic of metabolic syndrome is the maintenance of a steady state of mild and chronic inflammation [44], which significantly accelerates the exacerbation of the syndrome compared to that induced by each contributing disease.

#### 3.2.1. Chronic Inflammation Induction and Persistence

Several studies have shown that chronic inflammation plays an important role in the development of metabolic syndrome. For example, the Insulin Resistance Atherosclerosis Study (IRAS) (*n* = 1008; participant age: 40–69 years) showed that chronic inflammation strongly contributes to insulin resistance in some cases [45]. Furthermore, an increase in the levels of inflammatory markers, such as C-reactive protein (CRP) and fibrinogen, and white blood cell count associated with abdominal obesity may contribute to insulin resistance in individuals with obesity. The role of CRP in cardiovascular events is believed to be independently associated with insulin sensitivity. However, the IRAS findings indicated a role for inflammation in healthy individuals with insulin resistance. Another study showed that inflammatory markers are strongly associated with the incidence of asymptomatic atherosclerosis in a population with obesity [46]. In a different study, impaired inflammatory conditions and fibrinolysis were shown to strongly promote the development of type 2 diabetes [47]. The coexistence of metabolic syndrome and hypercholesterolemia increased the levels of thrombin-activatable fibrinolysis inhibitor and plasminogen activator inhibitor (PAI)-1, both of which inhibit fibrinolysis. This mechanism may underlie the development of inflammation-mediated disorders caused by metabolic syndrome and hypercholesterolemia. Furthermore, insulin resistance and chronic inflammation are reportedly involved in the development of diabetic retinopathy, a microvascular complication of type 2 diabetes [48]. The blood adiponectin levels in patients with diabetic retinopathy are higher than those in healthy participants [48]. Furthermore, they are positively correlated with the severity of diabetic retinopathy in patients with type 2 diabetes, indicating the close association between adiponectin levels and the maintenance of insulin sensitivity. Hence, adiponectin may be actively involved in the severity of diabetic retinopathy and type 2 diabetes [48].

#### 3.2.2. Visceral Fat Formation

Visceral fat refers to the fat accumulated in the abdominal cavity of the body. Fat can accumulate as ectopic fat in the liver, stomach, intestines, and arteries. Among the types of fat formed by human adipocytes, approximately 85% of the total fat mass is subcutaneous, and the remaining 15% is visceral fat [49]. Since waist size and fat accumulation are correlated [50], an above-standard waist size increases the likelihood of visceral fat hypertrophy and health problems [51]. For example, high visceral fat levels have been shown to contribute to the development of insulin resistance [52]. Excess visceral fat accumulation is associated with a high risk of insulin resistance [53], type 2 diabetes [54], heart disease [55], and breast and rectal cancers [56]. In addition, visceral fat accumulation may be involved in the development of systemic metabolic dysfunction prior to the onset of type 2 diabetes and vascular disease [57]. Excess visceral fat accumulation increases the likelihood of early events in serious, life-threatening medical conditions, such as myocardial infarction and stroke. In fact, multiple inflammatory molecules are released from visceral fat formed from long-term fat accumulation. For example, molecules secreted by abnormally accumulated visceral fat induce inflammation and increase the risk of chronic diseases, such as atherosclerosis [58]. Molecules secreted at high levels from visceral fat, such as interleukin (IL)-6, IL-1β, PAI-1, and TNF-α, induce inflammation and accelerate the development of the abovementioned diseases [59]. In patients with cardiovascular disease (CVD), the induction of chronic inflammation by these molecules is likely to induce and promote arterial plaque formation. As plaques are composed of cholesterol and dead cells, they are fragile and grow gradually, eventually rupturing [60]. When the plaques disintegrate, blood clots present in the arteries cause the partial or complete blockade of blood flow. When this condition is induced in the coronary arteries, the supply of oxygen to the heart is reduced, thereby leading to myocardial infarction and, in severe cases, death.

#### 3.2.3. Induction of Insulin Resistance

Insulin is secreted by the pancreas in response to increased blood sugar levels and stimulates cells in various tissues to promote glucose uptake and utilization. However, in insulin resistance, insulin-stimulated adipocytes, muscle cells, hepatocytes, and other cells fail to appropriately respond to insulin, as the blood glucose levels are constantly high. A prolonged hyperglycemic condition induces pathological metabolic disorders [61]. Insulin resistance is a typical pathological condition associated with metabolic syndrome, the development of various diseases, such as heart disease [62], and cancer [63], both of which are leading causes of death worldwide, as well as NAFLD [64], polycystic ovarian syndrome [65], and Alzheimer’s disease [66]. Reportedly, the heart disease risk considerably increases with insulin resistance or metabolic syndrome [67]. In insulin resistance, which reportedly affects more than 32.2% of the population in the United States, the cells stop responding to insulin [68]. The glucose-processing potential of insulin varies among individuals and is related to their genetic background [69]. Insulin resistance is induced by decreased responsiveness of individual peripheral tissues, such as skeletal muscle, fat, and liver tissues, to the action of insulin. Decreased insulin sensitivity and impaired insulin secretion, both of which are characterized by chronic hyperglycemia, are the major pathophysiological characteristics of type 2 diabetes [70].

Elevation in the levels of TNF-α secreted by adipocytes in patients with obesity is associated with insulin resistance-induced impaired glycemic control [71]. Studies have shown that large quantities of free fatty acids suppress insulin action on cells [72,73]. The increased blood free fatty acid levels are caused by an increase in body fat resulting from excessive calorie intake. Weight gain and obesity due to excess food intake strongly induce insulin resistance [74], which is one of the pathological characteristics of metabolic syndrome and type 2 diabetes. Therefore, metabolic syndrome is sometimes referred to as insulin resistance syndrome [75]. The symptoms of insulin resistance include high plasma triglyceride levels, high blood pressure, abdominal fat, high blood glucose levels, and low HDL cholesterol levels [76].

#### 3.2.4. Induction of Vascular Endothelial Cell Damage and Development of Arteriosclerosis

Obesity-induced inflammation also causes vascular cell inflammation, thereby increasing the risk of vascular endothelial damage and atherosclerosis [77,78]. In particular, the excessive secretion of inflammatory cytokines, including TNF-α, and suppression of anti-inflammatory cytokines from abnormal adipocytes are the potential mechanisms underlying obesity-induced inflammation and endothelial dysfunction [79,80]. Furthermore, the anti-inflammatory cytokines adiponectin and omentin, which exert anti-arteriosclerotic effects, inhibit type 2 diabetes-induced arteriosclerosis. The reduced expression of these anti-inflammatory cytokines promotes arteriosclerosis in patients with type 2 diabetes [81,82]. Inflammation-induced stimuli also increase the synthesis and production of inflammatory cytokines, chemokines, ICAM-1, and VCAM-1 in vascular endothelial cells. The increased expression of ICAM-1 and VCAM-1 in endothelial cells promotes monocyte adhesion and induces monocyte/macrophage accumulation in vascular tissues. Monocytes/macrophages that accumulate in vascular tissues take up oxidized LDL in large quantities and form foam cells and atherosclerotic lesions [83]. Meanwhile, in individuals with obesity, TNF-α acts on the adipose tissue around blood vessels to promote the activity of oxidases, such as NAD(P)H oxidase, and generate large quantities of ROS, which inactivate nitric oxide (NO) produced by vascular endothelial cells and reduce its relaxing effects [84] on smooth muscle cells leading to vasoconstriction [85]. In fact, chronic type 2 diabetes reportedly leads to progressive cardiovascular metabolic disorders that significantly increase the mortality rate from heart disease in patients aged above 65 years [86].

## 4. Effect of Soybean and Isoflavones on Metabolic Syndrome: Epidemiological Studies

Randomized controlled trials have shown that soybean-based food intake affects metabolic syndrome [87]. When patients with metabolic syndrome consumed soybean-based food (equivalent to 30 g of soy protein/day) for 12 weeks, the parameters related to metabolic syndrome improved in 13 of the 26 patients. This study also reported a significant improvement in body weight and the levels of multiple lipid markers involved in the induction of arteriosclerosis, such as total and LDL cholesterol. Another study compared the effects of soy nut (soaked and roasted soybean) and soy protein intake in 75 women aged above 60 years with borderline metabolic syndrome [88]. The participants were randomly assigned to three groups of 25 participants each and fed soy nut (daidzein 47.6 mg/35 g, genistein 60.2 mg/35 g, and glycitein 9.45 mg/35 g), soy protein (textured soy protein: daidzein 38.5 mg/35 g, genistein 48.8 mg/35 g, and glycitein 8.9 mg/35 g), and control food for 12 weeks. The soy nut intake group showed significant improvement in multiple lipid profile parameters. Soy protein intake significantly reduced the levels of total cholesterol, insulin, and malondialdehyde (MDA), an indicator of oxidation. Therefore, isoflavones present in soybean may improve the lipid profile and oxidative stress in patients with metabolic syndrome-associated CVDs.

In a randomized crossover clinical trial, women with metabolic syndrome were fed soybean to examine its effects on inflammation and vascular endothelial function [89]. Forty-two postmenopausal women with metabolic syndrome were administered a control diet (DASH diet), soy protein diet, or soy nut diet for eight weeks each. Soy nut and soy protein intake reduced the production of NO, which induces vascular relaxation, by 9.8% (*p* < 0.01) and 1.7% (*p* = 0.10), respectively, compared to that in the control group. In addition, soy nut and soy protein intake reduced the serum levels of E-selectin, which is involved in the induction of arteriosclerosis, by 11.4% (*p* < 0.01) and 4.7% (*p* = 0.19), respectively, compared to that in the control group. Furthermore, compared to the control diet, the soy nut diet significantly reduced the inflammation-induced production of IL-18 (by 9.2%) (difference from control diet: *p* < 0.01). Conversely, the level of IL-18 reduced in response to soy protein intake, but not significantly. The expression of CRP, which is associated with inflammation, was also examined. Soy nut and soy protein intake reduced the CRP levels by 8.9% (*p* < 0.01) and 1.6% (*p* < 0.01), respectively, compared to those in the control diet group. NO was previously shown to improve vascular health by suppressing arteriosclerosis, exert anti-thrombotic effects, and promote vasodilation [90,91]. Therefore, the habitual intake of soybean may improve the condition of vascular endothelial cells and the metabolic syndrome-related CVD symptoms.

Another study investigated whether the effects of soybean on metabolic syndrome are mediated by soy protein or isoflavones [92]. The study investigated the effects of soy protein and isoflavone intake in Koreans without metabolic syndrome aged 40 years or older (=5509; 2204 men, 3305 women). The incidence of metabolic syndrome was investigated independently, and the relationship between soy protein and isoflavone intake and the onset of metabolic syndrome was analyzed. Soy protein and isoflavone intake was significantly inversely correlated with the incidence of metabolic syndrome in women, whereas this correlation was not significant in men. However, soy protein and isoflavone intake was significantly inversely correlated with the LDL cholesterol levels in both men and women. Furthermore, abdominal obesity and hypertension were inversely correlated with soy protein and isoflavone intake only in women and inversely associated with elevated triglyceride levels only in men. These results indicate that the habitual intake of soy protein and isoflavones may be associated with a reduced risk of metabolic syndrome and its constituent diseases in humans.

## 5. Effects of Isoflavones on Metabolic Syndrome and Its Constituent Diseases

### 5.1. Genistein and Daidzein Effects on Adipocytes

When individuals with obesity consumed genistein capsules (50 mg/day) for two months, their intestinal flora was altered, insulin resistance was improved, and skeletal muscle fatty acid oxidation was promoted [93]. In experiments in the mouse precursor adipocyte line 3T3-L1 and adipocytes isolated from mice with obesity, genistein regulated the expression of miR-222 and its target genes encoding BTG2 and adiponectin. Genistein was also shown to regulate lipid metabolism in individuals with obesity [94]. These findings indicate that genistein may break down accumulated fat in individuals with obesity, thereby suppressing insulin resistance. Similarly, when cultured 3T3-L1 cells were stimulated with genistein (50 and 100 μM) for 12 days, the lipid droplets that accumulated in the cells changed to a multilocular form, and the fat mass decreased [95]. In addition, in this experiment, genistein was found to regulate acetyl-CoA carboxylase, fatty acid synthase, fatty acid-binding protein 4 (Fabp4), hormone-sensitive lipase, and chemerin, which are characteristic molecules present in white adipocytes. This action of genistein was mediated by alterations in the transcripts of genes (C/EBPβ, peroxisome proliferator-activated receptor-γ co-activator-1α, sirtuin1) involved in adipocyte differentiation. Genistein was shown to suppress the expression of multiple mRNAs, such as resistin mRNA, and increase the expression of CD-137 and UCP1 mRNAs, which are characteristic of brown/beige adipocytes. Conversely, when visceral adipocytes isolated from humans were cultured and stimulated with genistein (50 to 200 μM) for 14 days, the pre-visceral adipocytes differentiated and transformed into brown adipocytes [96]. AMP-activated protein kinase (AMPK) signaling may be involved in the action of genistein on adipocyte browning and contribute to the maintenance of mitochondrial function. These results indicate that genistein may stimulate the differentiation of adipocytes into beige/brown adipocytes. In contrast, daidzein was shown to reduce the expression of genes encoding MCP-1 and IL-6, which were shown to be involved in inflammation in a co-culture system of 3T3-L1-derived adipocytes and RAW264 macrophages [97]. In the same study, daidzein exerted a contrasting effect by promoting the expression of attenuated adiponectin. These findings indicated that adipocyte stimulation by macrophages mimics the state of inflammation that commonly occurs in obesity [98]. Daidzein may inhibit this macrophage-induced obesity-type inflammation. This study also showed that peroxisome proliferator-activated receptor γ (PPARγ) is involved in the anti-inflammatory effect of daidzein.

In recent years, genistin, a glycoside of genistein, has been shown to reduce fat accumulation in 3T3-L1 cells when administered at 50 and 100 µM, similar to the effects of the aglycone form of genistein [99]. In 3T3-L1 cells treated with 50 and 100 μM of genistin, lipid accumulation was reduced by 21.7% and 69.2%, respectively, and fat accumulation was reduced by 37.2% and 81.9%, respectively. In addition, morphological observations revealed that both genistin and genistein reduced lipid droplet formation. Genistin and genistein also dose-dependently suppressed the increased protein expression levels of C/EBPα, PPARγ, and FABP4 during pre-adipocyte-to-mature adipocyte differentiation. Moreover, genistin and genistein reduced the mRNA expression of ATP citrate lyase, acetyl-CoA carboxylase 1, and fatty acid synthase, which are involved in lipid synthesis. These results indicate that genistin and genistein may inhibit fat production via similar mechanisms. Thus, genistin regulates AMPK/SREBP-1c signaling, reduces the C/EBPα, PPARγ, and adipocyte-binding protein 2/FABP4 levels, and inhibits adipogenesis. However, it is necessary to demonstrate the action of genistin in vivo, as its action on 3T3-L1 cells suggested its potential anti-obesity effects [99].

### 5.2. Effect of Genistein and Daidzein on Vascular Function

Previous studies have shown the vasodilatory effects of genistein. The ingestion of genistein by postmenopausal women induced endothelium-dependent vasodilation [100]. Sixty healthy postmenopausal women were administered genistein (*n* = 30; 54 mg/day) or placebo (*n* = 30) for six months. Genistein intake increased the nitrite/nitrate levels from 22 ± 10 μmol/L to 41 ± 10 μmol/L and reduced the plasma endothelin 1 levels from 14 ± 4 pg/mL to 7 ± 1 pg/mL. These results suggested that genistein likely induces endothelium-dependent vasodilation in postmenopausal women by regulating the production of NO and endothelin 1 released from vascular endothelial cells. In another study, 20 postmenopausal women with metabolic syndrome received a placebo or genistein (54 mg/day) for six months along with a Mediterranean-style diet [101]. Six months after treatment, flow-mediated dilation increased significantly in women in their fifties who received genistein compared to that in women who received the placebo. In addition, compared to the placebo, genistein significantly reduced the total blood cholesterol, triglyceride, homocysteine, and bisfatin levels. Genistein intake was also found to increase the blood adiponectin levels. A recent study on blood phytoestrogen levels in 143 women enrolled in the Women’s Ischemia Syndrome Evaluation (1996–2001) showed the relationship between genistein levels and the risk of cardiovascular events [102]. In women with clinically suspected ischemic heart disease, low genistein levels were associated with an increase in cardiovascular events after six years [102], indicating that genistein intake may affect vasodilation and reduce the incidence of coronary events.

The effects of genistein, daidzein, and 17β-estradiol on vascular endothelial cell function have been investigated using aortic rings isolated from spontaneously hypertensive rats (SHRs) and Wistar Kyoto rats [103]. Genistein and daidzein enhanced eNOS activity in vascular endothelial cells and blocked NADPH-stimulated ROS production. Studies conducted using cultured human aortic vascular endothelial cells revealed that genistein enhances NO production via the PKA/CREB/eNOS/NO signaling pathway and plays a beneficial role in preventing hypertension [104]. Recent studies have shown the importance of the NO-mediated inhibition of CVDs [105], suggesting that genistein and daidzein possibly inhibit CVDs by inducing NO production in vascular endothelial cells. In addition, genistein suppressed superoxide production and NOX4 expression via ox-LDL in human umbilical vein endothelial cells [106]. In another study, human umbilical vein endothelial cells were stimulated with genistein (10,100, and 1000 nM), cultured for 6 h and further stimulated with ox-LDL (50 mg/L) for an additional 24 h [107]. Ox-LDL stimulation induced the expression of E-selectin, P-selectin, MCP-1, and IL-8 in endothelial cells, which decreased depending on the concentration of genistein added. In addition, this study showed that genistein may be involved in lowering miR-155 and raising SOCS1 levels via the NF-κB signaling pathway. Ox-LDL is involved in foamy macrophage formation, vascular smooth muscle cell proliferation and migration, and collagen deposition, all of which are known to contribute to the development of atherosclerosis [108]. Therefore, genistein intake may reverse the ox-LDL-induced inflammation in vascular endothelial cells and prevent arteriosclerosis.

### 5.3. Effect of Genistein and Daidzein on Type 2 Diabetes

A large-scale study conducted in men and women in the United States examined the association between soy food and isoflavone intake and the risk of type 2 diabetes [109]. Isoflavone intake assessment was based on self-reported results and the risk of type 2 diabetes was based on data obtained from nurse health survey I (1998–2012, 63,115 women), nurse health survey II (1999–2013, 79,061 women), and the results reported by healthcare professionals (2002–2010, 21,281 men). The total isoflavone intake and daidzein and genistein intake were found to be inversely correlated to the risk of type 2 diabetes.

Numerous effects of genistein and daidzein on type 2 diabetes have been reported. Some of them are summarized in Table 3 and discussed in the following paragraphs. First, the effects of genistein on type 2 diabetes and those related to type 2 diabetes will be described based on the results of human intake. The effect of genistein intake on oxidative stress, a metabolic parameter in postmenopausal women with type 2 diabetes, was investigated in 54 participants with type 2 diabetes (age: 47–69 years). The study population was divided into the genistein (*n* = 28, 54 mg) and placebo (*n* = 26) groups and genistein or placebo was administered twice daily for 12 weeks [110]. Genistein intake significantly reduced the levels of fasting blood glucose, glycated hemoglobin A1c, triglycerides, and serum MDA, and conversely, increased the total antioxidant capacity. In addition, genistein intake significantly increased the insulin sensitivity. In another randomized, double-blind, controlled trial, patients with NAFLD (*n* = 41) received 250 mg genistein daily for eight weeks. Compared to the placebo, genistein significantly reduced the serum insulin levels (*p* = 0.001) and significantly improved insulin resistance (homeostasis model assessment of insulin resistance (HOMA-IR), *p* = 0.041) [111]. Furthermore, genistein intake reduced the serum MDA, TNF-α, and IL-6 levels. Moreover, compared to the placebo, genistein significantly reduced the waist-to-hip ratio (*p* = 0.021), body fat percentage (*p* = 0.015), and triglyceride levels (*p* = 0.018). However, the body mass index and fasting blood glucose levels did not significantly change. The meta-analysis of data from randomized controlled trials showed the effects of genistein on glycemic control and insulin sensitivity [112]. The meta-analysis analyzed data from seven randomized controlled trials with a total study population of 670 individuals. Compared to the placebo, genistein significantly reduced the fasting blood glucose levels (*p* = 0.005). Furthermore, genistein has been shown to significantly improve glycemic control and insulin sensitivity in postmenopausal women. In another study, 120 postmenopausal women with metabolic syndrome were administered 54 mg genistein (*n* = 60) or placebo (*n* = 60) for one year to improve blood glucose levels, fasting insulin levels, and HOMA-IR [113]. In another study, the effects of daidzein and genistein on glycemic control and insulin sensitivity were investigated in 165 Chinese women with impaired glucose regulation. Neither daidzein nor genistein (both administered at 50 mg) exerted significant effects on glycemic control and insulin sensitivity in these women after six months of intake [114]. These results indicated that genistein may exert a beneficial effect on blood glucose levels and suppress the development of type 2 diabetes in humans, but further studies are required to clarify these discrepant results.

A study performed on a mouse model of obesity and diabetes showed that genistein blocked the induction and elevation of blood glucose levels [115]. Genistein ingestion (250 mg/kg diet) by the obese diabetic mice prevented the increase in blood glucose levels and improved glucose tolerance and blood insulin levels. However, it did not affect weight gain, food intake, fat deposition, the plasma lipid profile, or peripheral insulin sensitivity. In addition, genistein promoted islet β-cell survival by increasing the number of islet insulin-positive β cells and blocking apoptosis. These results suggested that besides improving blood glucose levels, genistein may act on β cells to directly protect them and prevent type 2 diabetes. Another study conducted in mice with high-fat diet- and streptozotocin-induced type 2 diabetes, showed that genistein administration (20 and 40 mg/kg) for eight weeks [116] reduced hyperglycemia, hyperlipidemia, and the serum levels of pro-inflammatory factors. Furthermore, genistein improved liver dysfunction, suppressed pathological changes, reduced the expression of inflammation-related proteins, and blocked hepatocyte apoptosis. Moreover, it ameliorated colonic pathological changes, the expression of tight junction-associated proteins, and the increase in pro-inflammatory factors. High concentrations of genistein alter the gut microbiota by increasing the levels of short-chain fatty acids to improve inflammation and insulin resistance. In another study, the administration of genistein (20 mg/kg/day) to alloxan-treated diabetic rats significantly reduced (*p* < 0.01) the alloxan-induced increase in blood glucose levels [117]. Genistein effectively improved insulin resistance, as observed in HOMA-IR assessments. In another study, genistein reduced the alloxan-induced increase in the levels of inflammatory cytokines, such as TNF-α, CRP, and IL-6. Moreover, it suppressed the alloxan-induced increase in triglyceride and LDL levels, and conversely, increased the serum HDL cholesterol levels. These findings suggested that genistein may stimulate insulin secretion by enhancing alloxan-stimulated GLP-1 secretion. Another experimental study showed that genistein blocked fructose-induced oxidative stress and inflammation in the serum and liver to improve the HOMA-IR levels and lipid status [118].

**Table 3:** Diabetes ameliorating effects of daidzein and genistein reported in epidemiological and animal studies.

Isoflavone	Study Types	Daidzein and/or Genistein Considered	Study Design and Conditions	Evidence	Relation *	Reference
Genistein	Epidemiological	The genistein group (*n* = 28.54 mg/capsule) and the placebo group (*n* = 26) took two capsules daily for 12 weeks.	54 postmenopausal women with type 2 diabetes between the ages of 47 and 69.	It improved genistein intake, T2DM postmenopausal women’s fasting blood glucose, glycated hemoglobin, serum TG, total antioxidant capacity and MDA.	+	[110]
		Randomly assigned to two. (1) Placebo; (2) Genistein 54 mg.	120 postmenopausal women with metabolic syndrome (placebo, *n* = 60; genistein 60)	Genistein intake improved the risk of T2DM and CVD in postmenopausal women with metabolic syndrome.	+	[113]
		Genistein 250 mg (*n* = 41), placebo (*n* = 41) taken daily for 8 weeks.	82 NAFLD patients	Improved fat metabolism, insulin resistance, oxidation and inflammatory index reduction in NAFLD patients.	+	[111]
	Animal	Genistein (20 and 40 mg/kg), 8 weeks forced intake.	High-fat diet/streptozotocin injection in C57BL/6J mice.	Metabolic disorders of glucose and lipids, improved dysfunction of liver and colon. Changed intestinal flora and improved inflammation and insulin resistance.	+	[116]
		Genistein (20 mg/kg/day)	30 Alloxan-induced diabetic rats.	Improves the harmful effects of alloxans on the pancreas and intestines. GLP-1 secretory stimulation.	+	[117]
		(1) Solid diet + genistein (0.25 mg/kg/day/rat); (2) Solid feed + fructose (20% fructose); (3) Solid feed + fructose (20%) + genistein (0.25 mg/kg/day/rat).	Oxidative stress and inflammation of rat serum and liver due to fructose.	Genistein improved antioxidant, anti inflammatory, HOMA-IR and lipid status in fructose-treated rats.	+	[118]
		Genistein (250 mg/kg meal).	High-fat diet/streptozotocin injection in C57BL/6J mice.	Improves hyperglycemia, glucose tolerance and blood insulin levels. Promotion of islet β-cell survival.	+	[115]
Daidzein	Epidemiological	Randomly assigned to three. In addition to 10 g of soy protein daily, (1) Placebo; (2) 50 mg of daidzein, and (3) 50 mg of genistein are administered for 24 weeks.	165 impaired glucose regulation Chinese women aged 30–70.	Daidzein and genistein have no significant effected on glucose control and insulin sensitivity.	−	[114]
	Animal	Daidzein (50 mg/kg daily) is forced oral administration for 12 weeks.	Ovarian ablation rats (12 weeks old).	Level weight gain, visceral fat accumulation, blood lipid, TNF-α, leptin, IL-6 level reduction.Improved insulin resistance (with HOMA-IR).	+	[119]
		Daidzein (0.1% in the diet)	L6 myotubes, db/db mice. KK-Ay mouse	Promotes glucose uptake, AMPK phosphorylation, and GLUT4 translocation. Improvement of gastrocnemius AMPK phosphorylation.	+	[120]
		Daidzein (25, 50, 100 mg/kg), 28 days.	Streptozotocin injection in C57BL/6J mice.	Protects retinal damage due to hyperglycemia. Preventive effects on diabetic retinopathy.	+	[121]

Abbreviations: CVD, cardiovascular disease; T2DM, type 2 diabetes mellitus; GLP-1, glucagon-like peptide 1; GLUT4, glucose transporter 4; HOMA-IR, homeostasis model assessment of insulin resistance; IL-6, interleukin-6; NAFLD, nonalcoholic fatty liver disease; MDA, malondialdehyde; TG, triacylglycerol; TNF-α, tumor necrosis factor-alpha. * +, positive relationship between isoflavone intake and reduced type 2 diabetes and type 2 diabetes-related pathologies.; −, no negative or no evident relation between isoflavone intake and decreased type 2 diabetes and type 2 diabetes-related pathologies.

The effects of daidzein on diabetes have been less frequently reported than those of genistein. The ingestion of daidzein (50 mg/kg daily) improved insulin resistance in ovariectomized rats [119]. In contrast, ovariectomy increased the body weight and visceral fat in rats, while daidzein intake decreased the HOMA-IR index and TNF-α and IL-6 levels along with the body weight and visceral fat. However, studies performed using L6 muscle cell lines and db/db mice showed that daidzein inhibited diabetes by promoting glucose uptake by translocating glucose transporter 4 [120]. Insulin resistance and chronic inflammatory stimuli strongly increase the risk of diabetic retinopathy development due to type 2 diabetes [48]. The effects of daidzein on diabetic retinopathy have been reported. In one study, rats with streptozotocin (55 mg/kg)-induced diabetes were administered daidzein (25, 50, and 100 mg/kg) for 28 days [121]. The results showed that diabetic control rats had significantly high levels (*p* < 0.001) of plasma glucose and plasma lactate dehydrogenase (LDH), an indicator of tissue damage. Conversely, the intake of 100 mg/kg daidzein significantly reduced the elevated blood glucose and LDH levels (*p* < 0.01). In addition, electroretinography and histopathological findings showed that the ingestion of 100 mg/kg daidzein reduced retinal thickening, suggesting that daidzein intake is likely to provide protection against hyperglycemia-induced retinal damage and effectively prevent diabetic retinopathy.

### 5.4. Effects of Genistein and Daidzein on Hyperlipidemia

Impaired lipid and lipoprotein metabolism is associated with the development of conditions that lead to metabolic syndrome, such as type 2 diabetes and CVDs [122]. The effect of isoflavones on dyslipidemia has been reported in different studies. Compared to placebo, genistein (60 mg/day) administration to 160 women with postmenopausal hyperlipidemia significantly reduced the triglyceride, total cholesterol, and LDL cholesterol levels (*p* < 0.05) [123]. In the same study, genistein increased the mRNA and protein expression levels of HDL cholesterol, LDL receptor, liver X receptor α and ATP-binding cassette transporter G1. Thus, genistein is likely to regulate the expression of the genes encoding these proteins and participate in cholesterol homeostasis. In another study, insulin signaling increased in ovariectomized rats fed a high-fat diet (45% fat) following genistein administration (15 or 30 mg/kg, via gavage, once daily) for four weeks. Furthermore, genistein reduced weight gain and prevented obesity in these rats and improved the insulin sensitivity of peritoneal white adipose tissue by increasing the IRS1 and p-AKT levels [124]. In particular, genistein increased the expression of UCP-1 and stimulated or promoted the browning of white adipocytes. Another study showed that in the liver cell line HepG2, genistein (≥1 μM) increased the intracellular cholesterol levels by inhibiting the PPARγ/LXRα/ABCA1 pathway via the SREBP-2/LDLR/HMGCR pathway [125]. Hence, the effect of genistein on the intracellular cholesterol level effectively reduced the plasma cholesterol levels and helped prevent atherosclerosis [125]. Mouse muscle C2C12 cells were used to investigate the effect of daidzein on fatty acid metabolism in muscle cells [126]. The stimulation of C2C12 cells with 50 μM daidzein induced the expression of pyruvate dehydrogenase kinase 4, acyl coenzyme A dehydrogenase, ATP synthase F1 subunit beta, and cytochrome c associated with fatty acid oxidation via ERRα. In addition, the expression of other genes, such as those involved in oxidation and oxidative phosphorylation was significantly promoted. These results indicated that genistein induced the expression of these genes in muscle cells to suppress lipid accumulation in muscles. The effects of daidzein and genistein on lipid metabolism were compared in rats [127]. The ingestion of daidzein (150 mg/kg diet) significantly reduced the serum and hepatic total cholesterol levels compared to those in the control group. Conversely, genistein did not affect the lipid levels; instead, it affected the expression of genes involved in cholesterol metabolism. Therefore, daidzein and genistein may affect lipid regulation through different mechanisms of action.

### 5.5. Effects of Genistein and Daidzein on Hypertension

A systematic review and meta-analysis of randomized controlled trials analyzed the effects of genistein intake on systolic blood pressure (SBP) and diastolic blood pressure (DBP) [128]. Four randomized controlled trials reported no significant reduction in SBP and DBP. Recent reports have shown that isoflavones may act on vascular endothelial cells to induce relaxation of vascular smooth muscle cells via multiple signaling pathways, thereby improving hypertension [129]. The effects of genistein on NO synthesis were investigated in primary cultures of human aortic endothelial cells and human umbilical vein endothelial cells [130]. Genistein (1–10 μM) activated eNOS in both cell types to increase NO synthesis. In addition, the administration of genistein to SHRs increased the aortic eNOS levels and aortic wall thickness, and reduced hypertension. In another animal-based study, the blood pressure-lowering and vasodilatory effects of daidzein sulfate, a water-soluble derivative of daidzein, were demonstrated in SHRs. The administration of daidzein sulfate (at 20 and 40 mg/kg body weight) lowered the blood pressure of SHRs [131]. The antihypertensive effect of daidzein sulfate was attributed to the suppression of extracellular Ca^2+^-dependent contraction. This suggests that daidzein sulfate exerts strong blood pressure-lowering and vasodilatory effects, primarily on arterial smooth muscle cells. In summary, although there are some inconsistencies in the reported effects of isoflavones in humans, evidence from studies published until 2020 demonstrates that dietary supplements, including soy isoflavones (present in beverages such as beetroot juice, which is also rich in magnesium, vitamin C, and catechins), may help lower blood pressure.

## 6. Conclusions

**Figure 3:**
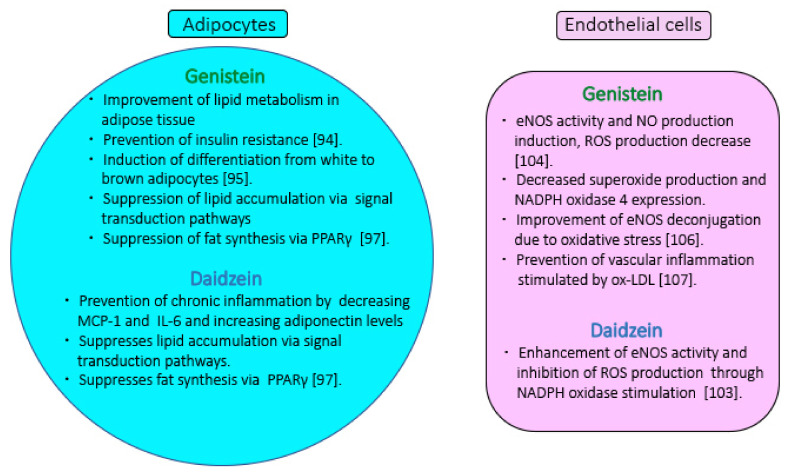
Preventive effects of isoflavones on vascular endothelial cells and adipocytes involved in metabolic syndrome. In adipocytes, daidzein blocks inflammation and suppresses fatty acid synthesis [97]. Genistein promotes lipid synthesis, lipid accumulation, and improves lipid metabolism [94,95,97]. Genistein promotes the induction of differentiation into brown adipocytes [95,97]. In vascular endothelial cells, daidzein and genistein enhance the activity of eNOS and block ROS production [104,106]. Genistein improves eNOS uncoupling due to oxidative stress. Genistein inhibits ox-LDL-induced vascular inflammation [107]. Abbreviations: MCP-1, monocyte chemoattractant protein-1; IL-6, interleukin-6; PPAR, peroxisome proliferator-activated receptor; eNOS, endothelial nitric oxide synthase; ROS, reactive oxygen species; ox-LDL, oxidized low-density lipoprotein.

This review sought to assess the effects of soy isoflavones on metabolic syndrome. The results of epidemiological studies indicate that soy and isoflavone intake may affect the individual conditions that cause metabolic syndrome as well as the process of disease development. The anti-inflammatory properties of isoflavones reportedly play an important role in inhibiting the development of metabolic syndrome. In addition, several metabolic risk factors associated with the progression of metabolic syndrome have been identified, including an association between the obesity-induced increase in body fat and induction of insulin resistance. Metabolic syndrome and its contributing diseases are strongly associated with obesity, adipocyte hypertrophy, and changes in adipocytokine expression. Vascular endothelial cell dysfunction is characterized by the exacerbation of vasodilation owing to a decrease in NO synthesis in the arteries, which accelerates the induction of hypertension. Vascular endothelial cell dysfunction is an early cause of CVDs associated with metabolic syndrome. The effects of daidzein and genistein on metabolic syndrome are associated with their effects on the respective induction mechanisms of obesity, adipocyte formation, vascular dysfunction, diabetes, and hyperlipidemia. Figure 3 summarizes the action of isoflavones on vascular endothelial cells and adipocytes. In particular, the isoflavone-inducing action of adiponectin secreted from adipocytes is critical in inhibiting the formation of diabetes. Furthermore, soy isoflavone increases the health of vascular endothelial cells. The multiple and diverse properties of isoflavones, such as daidzein and genistein, exert a potential prophylactic effect on metabolic syndrome symptoms. These details are expected to be more clearly demonstrated in future studies on humans.

## Figures and Tables

**Table 1:** Primary components of soybean and legumes.

	Soy Beans	Azuki Beans	Chickpeas	Lentils	Pinto Beans
Energy (kcal/100 g)	446	329	378	352	347
Protein (g/100 g)	36.49	19.87	20.47	24.63	21.42
Carbohydrate (g/100 g)	30.16	62.90	62.95	63.35	62.55
Fats (g/100 g)	19.94	0.53	6.04	1.06	1.23
Fiber (g/100 g)	9.3	12.7	12.2	10.7	15.5
PUFA (g/100 g)	11.255	0.113	2.731	0.526	0.407
Iron (mg/100 g)	15.70	4.98	4.31	6.51	5.07
Calcium (mg/100 g)	277	66	57	35	113

PUFA: polyunsaturated fatty acids. Source: Revised from reference Rizzo and Baroni, 2018 [25].

**Table 2:** Isoflavone content of soybean and processed soybean products.

SOYBEANS and Their Processed Products	mg per 100 g
Soy beans, raw	154.53
Soy beans, roasted	148.5
Tofu	13.1~34.78
Soy milk	0.7~10.73
Miso	41.45
Natto	82.29
Shoyu (soy sauce)	1.18
Edamame	17.92
Tempeh	3.82
Okara	9.39
Soybean oil	0

Source: Revised from reference Rizzo and Baroni, 2018 [25].

## Data Availability

Not applicable.
